# Estimating extra length of stay due to healthcare-associated infections before and after implementation of a hospital-wide infection control program

**DOI:** 10.1371/journal.pone.0217159

**Published:** 2019-05-17

**Authors:** Habibollah Arefian, Stefan Hagel, Dagmar Fischer, André Scherag, Frank Martin Brunkhorst, Jens Maschmann, Michael Hartmann

**Affiliations:** 1 Center for Sepsis Control and Care (CSCC), Jena University Hospital, Jena, Germany; 2 Department of Pharmaceutical Technology and Biopharmacy, Friedrich-Schiller University Jena, Jena, Germany; 3 Hospital Pharmacy, Jena University Hospital, Jena, Germany; 4 Institute for Infectious Diseases and Infection Control, Jena University Hospital, Jena, Germany; 5 Institute of Medical Statistics, Computer and Data Sciences, Jena University Hospital, Jena, Germany; 6 Center for Clinical Studies, Jena University Hospital, Jena, Germany; 7 Paul-Martini-Clinical Sepsis Research Unit, Department of Anesthesiology and Intensive Care Medicine, Jena University Hospital, Jena, Germany; 8 Jena University Hospital, Jena, Germany; University of Maryland School of Medicine, UNITED STATES

## Abstract

**Introduction:**

Healthcare-associated infections (HAIs) are a major health concern and have substantial effects on morbidity and mortality and increase healthcare costs. We investigated the effect of a hospital-wide program for the prevention of HAIs on additional length of stay (LOS).

**Methods:**

We analyzed data from a prospective, single-center, quasi-experimental study with two surveillance periods before and after implementation of an infection prevention intervention program. HAI diagnosis was made according to surveillance definition criteria established by the US Centers for Disease Control and Prevention. A multistate model was used to estimate additional LOS for patients with HAI in both surveillance periods.

**Results:**

During the first and second periods, 1,568 and 2,336 HAIs were identified among 26,943 and 35,211 patients, respectively. For HAI patients exclusively treated in a general ward, additional LOS was 8.4 (95% confidence interval, CI: 6.8–10.0) days in the first period and 9.6 (95% CI: 8.3–11.0) days in the second period (p = 0.26). For HAI patients treated in both an intensive care unit (ICU) and a general ward, additional LOS was 8.1 (95% CI: 6.3–9.9) days in the first period to 7.3 (95% CI: 6.1–8.5) days in the second period (p = 0.47).

**Conclusions:**

Healthcare-associated infections prolong LOS. A hospital-wide infection control program did not alter the prolongation of LOS.

## Introduction

Healthcare-associated infections (HAIs) are a major health concern and have substantial effects on morbidity and mortality[1]. HAIs prolong patients’ length of stay (LOS) and increase the costs of treatment [2]. Minimizing HAIs, particularly within hospitals, is a key aspect of patient safety initiatives in many countries, including Germany. The World Health Organization reported an estimated number of 5 million HAIs in acute care hospitals in Europe, resulting in 50,000 deaths and €13–24 billion in extra costs per year[3].

Because the cost of HAIs is strongly dependent on patients’ LOS, many studies have estimated additional LOS due to HAIs. Additional hospital stay must be calculated to assess how many bed-days might be gained from prevention measures[4]. Generally, most health economic studies have not considered the time of occurrence of HAIs and temporal dynamics of the individual patient (from hospital admission to discharge alive or death) in the estimation of additional LOS. Thus, the additional LOS and extra costs resulting from HAIs may be overestimated [5, 6]. Multistate models have been recommended to eliminate this time-dependent bias and estimate the extra time spent in the hospital due to HAIs using transition probabilities[7]. Such models define events over the course of time as transitions between various states, and the transition hazards are the main statistical quantities[8]. An intervention prevention program is an effective process to decrease the number of HAIs. Previous studies have shown that the implementation of an infection prevention program has benefits for patients and can reduce the numbers of HAI [9, 10]. Furthermore, we know that the severity of HAI is influenced by an infection control program and HAI management [11]. However, it remains unclear whether a reduction of severe HAIs can also influence extra LOS due to HAIs.

Therefore, we hypothesised that the extra length of stay associated with HAIs can be changed with the help of an infection prevention intervention program. We aimed to assess the impact of HAIs on LOS using multistate models in the surveillance period 1 and the surveillance periods 2 of a hospital-wide infection control program at Jena University Hospital.

## Materials and methods

### Study design

We analyzed data on HAIs from the ALERTS study [11], a prospective, quasi-experimental study at Jena University Hospital, a tertiary care medical center in Germany. The data protection commissioner and institutional review board approved the study with a waiver of informed consent for individual patients (ID: 3139-05/11). The trial has been registered at the German Clinical Trials Register (DRKS00003166) and the protocol was approved by the ethics committee of the Medical Faculty of the Friedrich-Schiller-University Jena. The need for consent was waived by the ethics committee. The TREND statement checklist was used for the reporting of non-randomized trials (S1 Table). The design of the ALERTS study included 1) a 12-month first surveillance period that was performed between September 2011 and August 2012; 2) a multifaceted hospital-wide program for infection control starting in October 2012; and 3) a second surveillance period to evaluate the effectiveness of the intervention, which was implemented from May 2013 to August 2014. Note that we previously published data on additional LOS and economic costs for the first surveillance period [2].

The multifaceted intervention program for infection control in the ALERTS study involved the hospital-wide promotion of hand hygiene and the implementation of prevention bundles for specific HAIs (S1 Fig). This intervention started in October 2012 and was conducted throughout the remainder of the study period. HAI were identified among patients hospitalized for ≥48 hours with at least 1 risk factor for HAI and new antimicrobial therapy. HAI diagnosis was made according to surveillance definition criteria established by the US Centers for Disease Control and Prevention[12]. All inpatients with a hospital stay ≥ 48 hours admitted to one of the wards under surveillance were eligible for inclusion. Wards under surveillance included 11 internal medicine wards (336 beds), 9 surgical wards (250 beds), 2 geriatric wards (39 beds), 2 neurological wards (50 beds), 3 gynecological wards (51 beds) and 5 intensive care units (ICUs)/intermediate critical care units (91 beds). Further details of the ALERTS study have been described elsewhere[11].

### Length of stay

A multistate model was used to estimate additional LOS due to HAIs considering HAIs as a potential intermediate state between admission and discharge or death to address the time-dependent bias.

**Fig 1:**
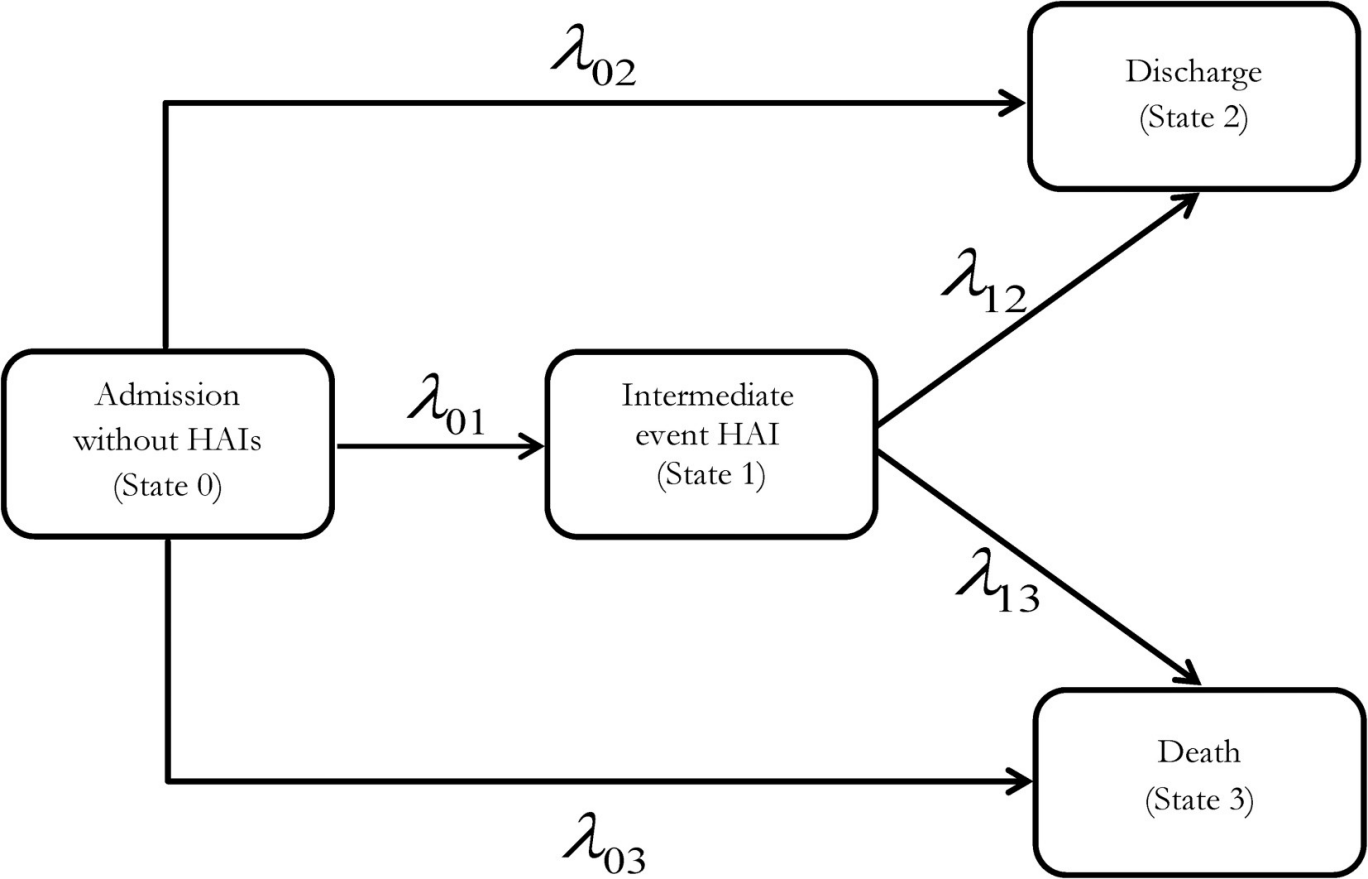
Multistate model with four states. Admission (state 0) is the first state, and all patients entered into the initial state without HAIs. The patient may acquire an HAI and move to intermediate state 1. Discharge (state 2) or death (state 3) indicates the end of hospitalization.

In this model, all patients were regarded as “non-exposed patient” as long as they did not acquire an HAI during their stay in the hospital. If a patient acquired more than one HAI, the time of the occurrence of the first HAI was used in the multistate model. The prolongation of LOS was derived as a function of the hazard of transition in the next short time between the health states[2, 5, 13]. Using the general multistate model displayed in Fig 1, we estimated the prolongation of LOS using two approaches. Model approach 1 estimated additional LOS due for the following HAIs (jointly and stratified) separately for the first and second surveillance periods: surgical site infection (SSI), lower respiratory tract infection (LRTI), urinary tract infection (UTI), primary blood stream infection (BSI), *Clostridioides difficile*-associated infection (CDI), other infections, and multiple infections (grouped under the category “Multiple”). Model approach 2 estimated the prolongation of LOS associated with HAIs stratified by department because the costs of bed-days largely differ across departments. Moreover, from the health economic perspective is important to know the extra LOS explicitly for patients stayed in ICU, because the daily costs of intensive care are higher than the treatment costs in general wards. Therefore, we estimated additional LOS separately for patients treated in general wards (surgical, internal medicine, geriatric, gynecology and neurology) and for patients treated in both a general ward and an ICU. The department or unit that discharged the patient from the hospital was considered as the main ward.

### Statistical analyses

In this study, we used multistate models that describe the daily risk of transition between multiple health states. The expected LOS associated with HAI (in days) was computed by a function of these transition probabilities[8]. The Aalen-Johnson estimator was used as a nonparametric estimator for the matrix of transition probabilities for all observed transition times[14]. We calculated the standard error (SE) and confidence intervals (CIs) for extra LOS by bootstrap sampling using 1,000 replicates. Additional LOS due to HAIs in the two surveillance periods were compared by using the z-test. The statistical tests were 2-sided and p-values of ≤0.05 were considered statistically significant.

In addition, we used the landmark method[15] to evaluate whether HAIs prolonging effect. We selected a range of landmark time points (s) for the analysis, s > 2. Given HAI status at time s, we then compared the probabilities of having reached the absorbing states 2 (discharge alive) and 3 (death) by time t, s ≤ t. We computed probability estimates within HAI groups defined at time s, taking s as the new time origin. In our multistate setting, we utilized the Aalen-Johansen estimator for patients discharged alive [P_02_(s, t) and P_12_(s, t)] and for death [P_03_(s, t) and P_13_(s, t)] for different landmark time points (s)[16].

All statistical analyses were performed using R version 3.2.3 (R Foundation for Statistical Computing), including the etm 0.6–2, mvna 2.0, and kmi packages[16].

## Results

**Fig 2:**
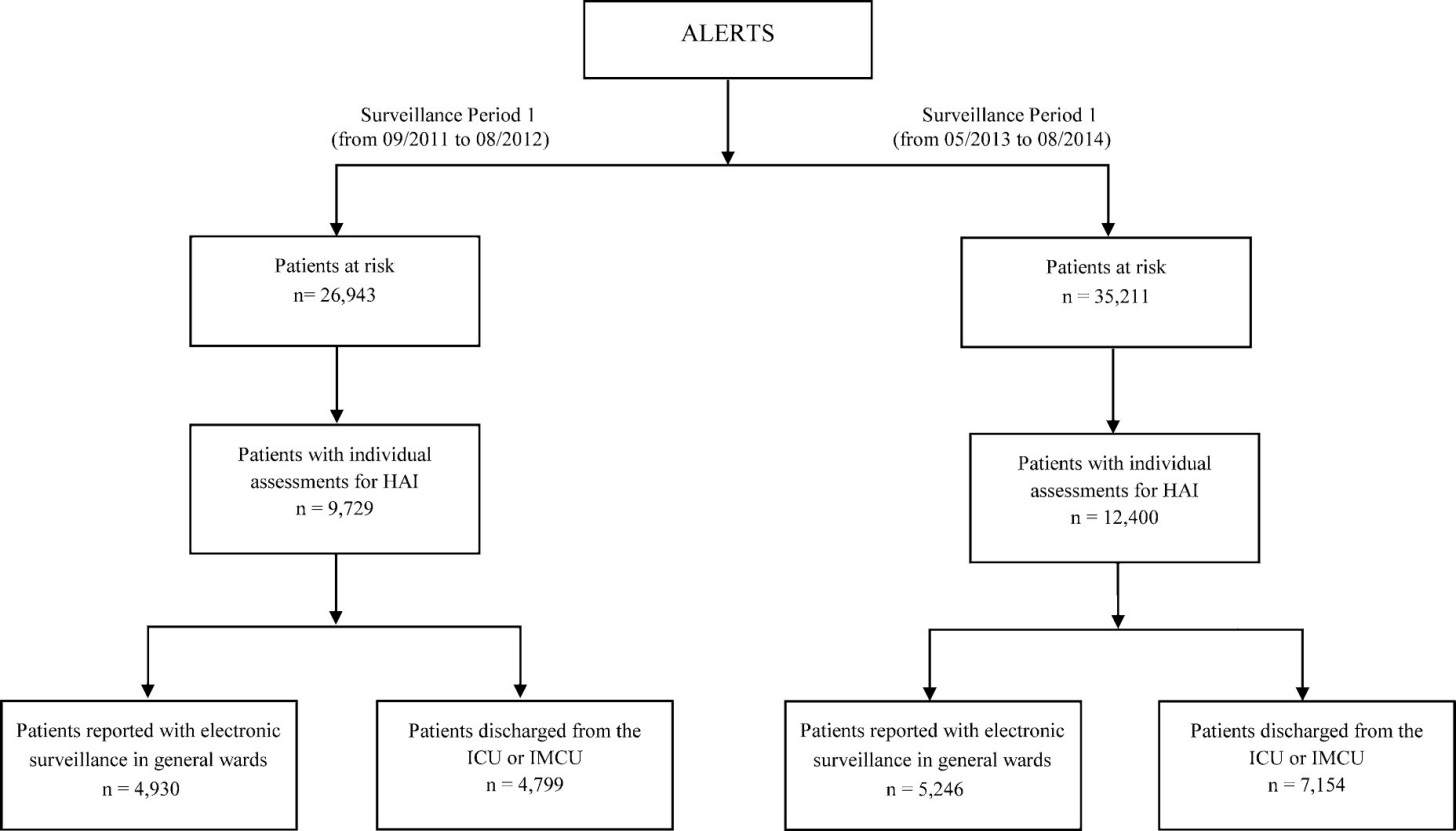
Flow chart of surveillance of healthcare-associated infections (HAIs) in ALERTS study.

During the ALERTS study, 26,943 patients in the first and 35,211 patients in the second surveillance period were admitted to the Jena University Hospital (Fig 2). In total, 1,170 patients in the first and 1,711 patients in the second surveillance period experienced 1,568 and 2,336 separate episodes of HAIs, respectively. The percentage of patients with HAI was 4.3% in the first surveillance period and 4.9% in the second surveillance period. The incidence of HAIs did not significantly differ between the first and second surveillance periods in general wards (Incidence rate ratio: 1.296; 95% CI, 0.784–2.145, p = 0.312) and in ICU (Incidence rate ratio: 0.592; 95% CI, 0.267–1.310, p = 0.196). A detailed comparison of the HAIs in the two periods has been reported elsewhere[11].

The median LOS of patients included in our analysis was six days (interquartile range (IQR): 3–11) in the first surveillance period and seven days (IQR: 4–11) in the second surveillance period. The median LOS of patients with HAI was 28 days (IQR: 18–42) in both periods. In total, 893 (3.3%) and 1,191 (3.4%) of the included patients died in the hospital during the first and second surveillance periods, respectively (Table 1).

**Table 1:** Selected characteristics of patients in the ALERTS study (for details, see Hagel et al. publication[11]).

Characteristic	Value	
	Surveillance Period 1 (09/2011 to 08/2012)	Surveillance Period 2 (05/2013 to 08/2014)
Number of patients with HAI—no. (%)	1,170	1,711
- Patients with one HAI	899 (77%)	1,268 (74%)
- Patients with more than one HAI	271 (23%)	443 (26%)
Male sex—no. (%)	643 (55%)	983 (57%)
Age, median years (IQR)	69 (56–76)	69 (57–76)
Hospitalization in the previous 3 months—no. (%)	329 (28%)	447 (26%)
Patients with severe sepsis/septic shock—no. (%)	351 (30%)	434 (25%)
In-hospital deaths due to HAIs–no. (%)	113 (10%)	164 (10%)
Site of infection—no. (%)^a^	1,568	2,336
- Surgical site infection	448 (29%)	625 (27%)
- Respiratory tract infection	385 (25%)	682 (29%)
- Primary bloodstream infection	202 (13%)	278 (12%)
- Urinary tract infection	162 (10%)	309 (13%)
- *Clostridioides difficile*-associated infection	163 (10%)	176 (8%)
- Other	208 (13%)	266 (11%)

HAI, healthcare-associated infection; IQR, interquartile range.

^a^ The percentages are about site of infection

### Prolongation of length of stay using a multistate model

Overall, the additional LOS due to HAIs (regardless of the unit in which the patients stayed; Model approach 1) was 12.0 (95% CI: 10.9–13.2; SE: ± 0.6) days in the first surveillance period and varied by type of HAI from 3.3 (95% CI: 3.1–3.5; SE: ± 0.1) days for UTI to 12.9 (95% CI: 10.6–15.1; SE: ± 1.1) days for SSI. In the second surveillance period, the additional LOS was similar, with 12.3 (95% CI: 11.4–13.2; SE: ± 0.4) days, and varied by type of HAI from 5.3 (95% CI: 3.5–7.1; SE: ± 0.8) days for UTI to 13.2 (95% CI: 11.1–15.3; SE: ± 1.1) days for SSI. The additional LOS for patients with multiple HAIs was 25.6 ± 1.5 days and 25.4 (95% CI: 23.0–27.8; SE: ± 1.2) days during the first and second surveillance periods, respectively (Table 2).

**Table 2:** Model approach I: Results of additional length of stay estimates from the multistate model stratified by infection site/type and surveillance period.

Comparison	Surveillance period 1^a^ *Additional LOS in days (95% CI)*	Surveillance period 2^a^ *Additional LOS in days (95% CI)*	p-value^b^
Urinary tract infection	3.3 (3.1–3.5)	5.3 (3.5–7.1)	0.03
*Clostridioides difficile*-associated infection	6.1 (3.2–9.1)	6.8 (4.3–9.4)	0.73
Lower respiratory tract infection	8.8 (7.1–10.6)	9.0 (7.5–10.4)	0.92
Primary blood stream infection	12.5 (8.0–17.0)	9.3 (6.7–11.9)	0.22
Surgical site infection	12. 9 (10.6–15.1)	13.2 (11.1–15.3)	0.84
Other infections	6.0 (3.1–8.9)	7.1 (4.1–10.1)	0.59
Multiple infections	25.6 (22.6–28.5)	25.4 (23.0–27.8)	0.93
Total	12.0 (10.9–13.2)	12.3 (11.4–13.2)	0.71

CI, confidence interval; LOS, length of stay.

^a^ Surveillance period 1 was from 09/2011 to 08/2012 and period 2 was from 05/2013 to 08/2014

^b^ P-Value relate to surveillance period 1 versus surveillance period 2

The estimates of prolongation of LOS due to HAIs according to the multistate model examined by department (Model approach 2) showed that additional LOS among patients exclusively treated in a general ward was increased from 8.4 (95% CI: 6.8–10.0; SE: ± 0.8) days in the first period to 9.6 (95% CI: 8.3–11.0; SE: ± 0.7) days in the second period. By contrast, the additional LOS decreased from 8.1 (95% CI: 6.3–9.9; SE: ± 0.9) days in the first period to 7.3 (95% CI: 6.1–8.5; SE: ± 0.6) days in the second surveillance period for patients with HAIs treated in both a general ward and an ICU (Table 3). However, the prolongation of LOS due to HAIs did not significantly change between the first and second surveillance periods among patients exclusively treated in a general ward (p = 0.23) or in both a general ward and an ICU (p = 0.47).

**Table 3:** Model approach 2: Extra days of hospitalization due to healthcare-associated infections stratified by department (rows), clinical unit stay of the patient and surveillance period (columns).

Model	Extra days (95% CI); patients treated exclusively in a general ward			Extra days (95% CI); patients treated in a general ward and an ICU		
	*Surveillance period 1*^a^	*Surveillance period 2*^a^	p-Value^b^	*Surveillance period 1*^a^	*Surveillance period 2*^a^	p-Value^b^
Surgical	6.5 (3.2–9.8)	10.9 (7.3–14.5)	0.07	7.5 (5.2–9.7)	7.4 (5.8–9.1)	0.97
Geriatric	5.3 (1.6–8.9)	6.9 (4.2–9.5)	0.47	8.6 (1.5–15.8)	4.7 (-1.5–11.0)	0.41
Gynecology	6.7 (0.8–12.6)	9.2 (2.7–15.6)	0.57	11.3 (3.8–18.8)	4.8 (0.6–9.1)	0.13
Internal medicine	8.8 (6.7–11.0)	8.0 (6.3–9.6)	0.51	11.0 (6.8–15.2)	6.4 (4.0–8.7)	0.05
Neurology	21.3 (3.5–39.1)	8.2 (4.7–11.7)	0.15	6.9 (3.3–10.5)	7.3 (4.8–9.9)	0.84
Total	8.4 (6.8–10.0)	9.6 (8.3–11.0)	0.26	8.1 (6.3–9.9)	7.3 (6.1–8.5)	0.47

CI, confidence interval.

^a^ Surveillance period 1 was from 09/2011 to 08/2012 and period 2 was from 05/2013 to 08/2014

^b^ P-Value relate to surveillance period 1 versus surveillance period 2

**Fig 3:**
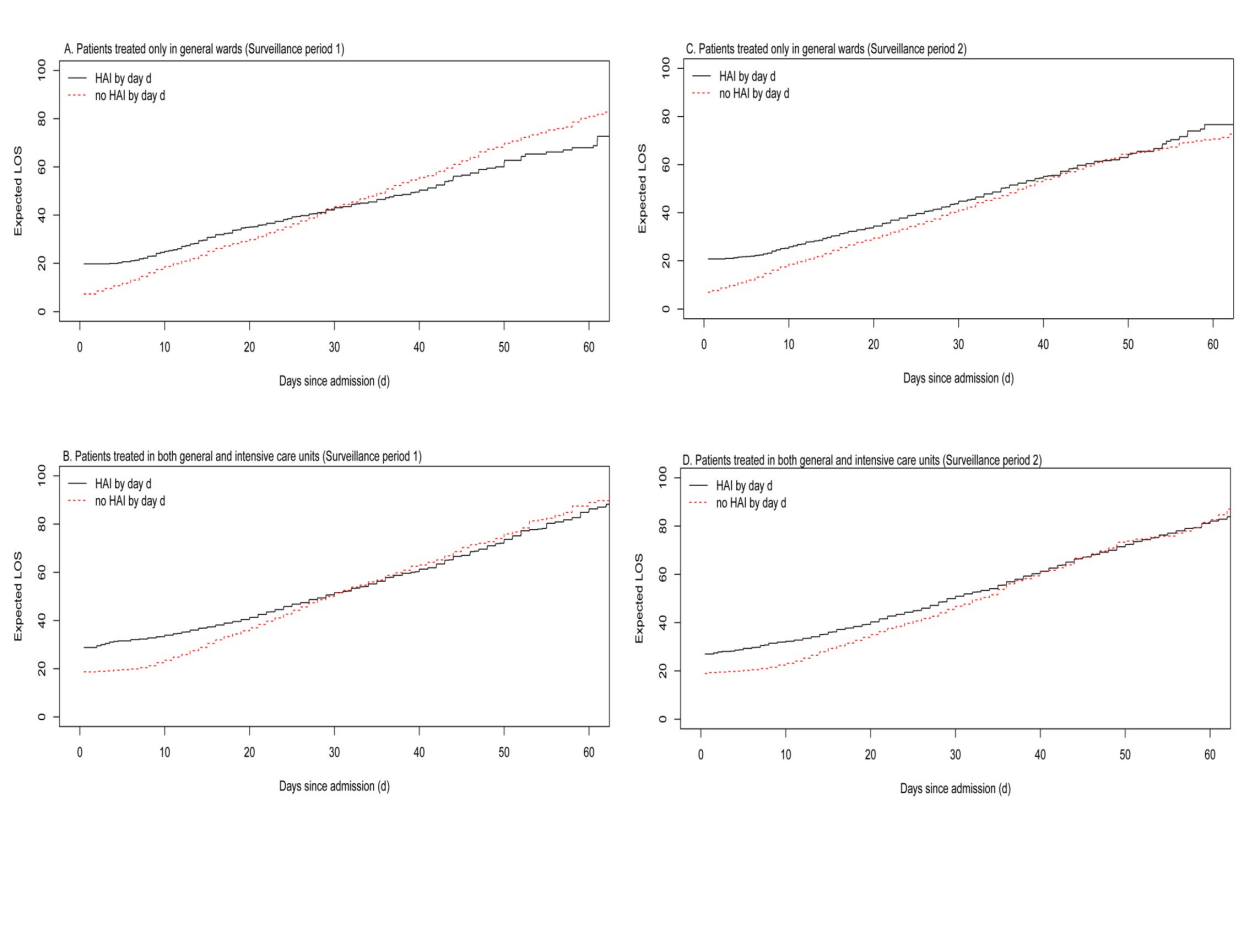
Results of multistate models to determine expected length of stay for patients with and without HAIs up to 60 days after admission.

The additional LOS due to HAIs was reduced in internal medicine departments from 11.0 (95% CI: 6.8–15.2; SE: ± 2.1) days in the first period to 6.4 (95% CI: 4.0–8.7; SE: ± 1.2) in the second period (P = 0.05) for patients who were treated temporary in ICU. Fig 3 illustrates the expected LOS on each day up to 60 days after admission using the multistate model for HAI patients versus non-HAI patients considering the patients treated only in a general ward versus those treated in both a general ward and an ICU in the first and second periods.

**Fig 4:**
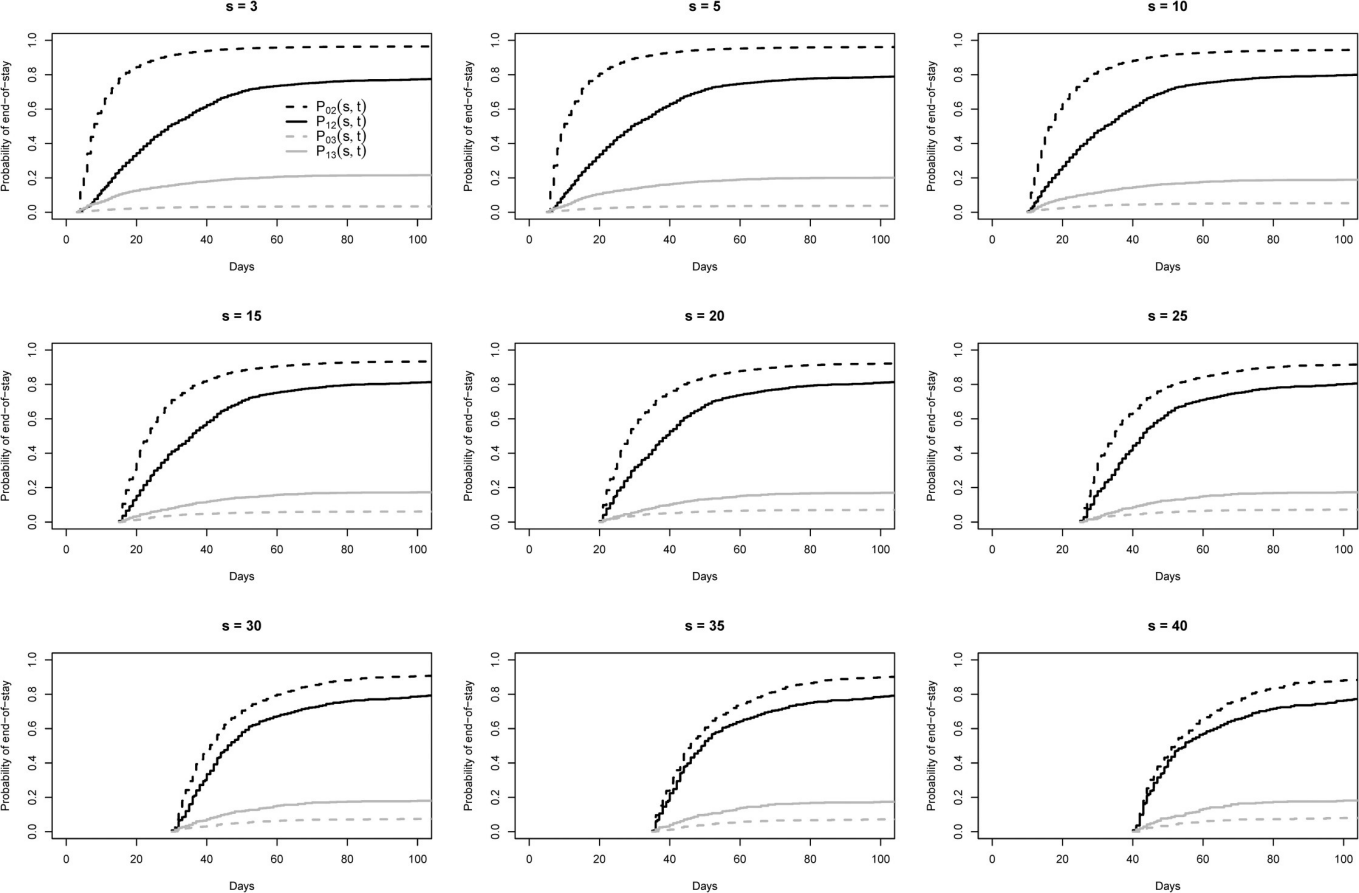
Results of the Aalen-Johansen estimator for patients discharged alive [P02(s, t) (black dashed lines) and P12(s, t) (black solid lines)] and for death [P03(s, t) (dark grey dashed lines) and P13(s, t) (dark grey solid lines)] for different landmark times s in surveillance period 1.

Our analysis for patients discharged alive [P_02_(s, t) and P_12_(s, t)] based on a range of landmarks s in the first surveillance period (Fig 4) showed that the prolonging effect of HAI is illustrated by the fact that, overall, P_02_(s, t) ≥ P_12_(s, t), meaning that the probabilities of discharged alive for patients without HAI are in different range of landmarks higher than for patients with HAI. The effect was most pronounced for early landmarks. Conversely, this analysis for death [P_03_(s, t) and P_13_(s, t)] for the same range of landmarks in the first period (Fig 4) showed that P_13_(s, t) ≥ P_03_(s, t), meaning that the probabilities of death for patient with HAI are higher than for patient without HAI in the same range of landmarks. The results were very similar in the second surveillance period (S2 Fig).

## Discussion

We estimated the impact of HAIs on prolongation of LOS during the first and second surveillance periods of the ALERTS study. Our analyses showed that the estimation of additional LOS due to HAIs based on the multistate model was sensitive to several factors, including the inpatient units and type of infection. For example, when patients were included in the multistate model based on ICU or non-ICU stay (model approach 2), the additional LOS due to HAIs was clearly lower than the estimated additional LOS associated with HAIs in model approach 1 when the inpatient units were ignored in analyses. The results also showed that prolongation of LOS due to multiple HAIs was 2 times greater than that due to SSI and 7 times greater than that due to UTI during the first surveillance period, and similar results were found in the second surveillance period.

However, the incidence of HAIs in the second surveillance period did not differ significantly after the intervention prevention program. Only a reduction in severe HAIs were observed in the second surveillance period in the ICUs[11]. Furthermore, our result shows that the additional LOS due to HAIs was reduced in internal medicine wards during in second surveillance period for patients who were treated some time in ICU. Infection prevention program for HAI cannot reduce additional length of stay due to HAI, but can change the ICU length of stay in some type of HAI. This is important to know because a reduction of LOS in intensive care unit can lead to substantial reduction in total inpatient cost[17].

A landmark method was applied to illustrate the prolonging effect of HAI for exposed patients in comparison with non-exposed patients. The results showed that the probabilities of discharged alive for patients with HAI were lower than patients without HAI at the same landmark. Furthermore, the probability of death for exposed patients is higher compared to non-exposed patients. Differences were higher at earlier landmark, meaning that the impact of occurrence of HAI in early days after admission on competing events (discharge alive, death) is much higher.

A systematic review of published literature concluded that additional LOS due to HAIs in studies using time-fixed methods was 9.4 days on average longer than that in studies using multistate models[18]. Therefore, our results are similar to those of other recent studies using a multistate model. For example, Stewardson et al. estimated that prolongation of LOS averaged 12.22 and 10.35 days for primary BSI with methicillin-resistant staphylococcus aureus and methicillin-susceptible staphylococcus aureus of acute care admissions at 10 European hospitals[19]. Macedo-Vinas et al. estimated a similar impact of HAIs at a tertiary care hospital in Switzerland[20].

This study has several limitations. We were unable to conduct an economic analysis because the infection control program did not reduce the incidence rate of HAIs. Our study analyzed data from a single-center study in Germany; thus, the results may not be generalizable to other settings. In addition, our data does not allow for an adjustment according to sex, age or comorbidities because of low number of patients with HAI in some categories.

The role of hand hygiene as the most important factor in the prevention of HAIs is accepted, and several studies have reported that the rate of adherence to hand hygiene can increase significantly with a multifaceted intervention, which can reduce the risk of HAIs[21]; however, the incidence of HAIs and extra LOS due to HAIs was not significantly reduced in the ALERTS study. One potential reason is that hand hygiene compliance is usually lower in some units, including ICUs, because of high activity level, lack of time and high workload[22, 23]. Moreover, hand hygiene compliance could have been affected by the Hawthorne bias in this study. Hagel et al. reported that hand hygiene events by healthcare workers can be increased from 8 to 21 events per hour because of the presence of a direct observer[24].

In conclusion, healthcare-associated infections prolong LOS and can therefore increase the hazard of hospital mortality and costs of care. Our analyses demonstrated that additional LOS varied across HAI sites. This information can help health policy makers determine which hospital infection prevention measures to invest in to improve health and save costs.

## Supporting information

S1 Fig Characterization of interventions for infection control in the ALERTS. (PDF)

S2 Fig Results of the Aalen-Johansen estimator for patients discharged alive [P02(s, t) (black dashed lines) and P12(s, t) (black solid lines)] and for death [P03(s, t) (dark grey dashed lines) and P13(s, t) (dark grey solid lines)] for different landmark times s in surveillance period 2. (PDF)

S1 Table TREND statement checklist. (PDF)

S1 Dataset Copy of dataset. (XLSX)
