# Supplementary material for: Estimating extra length of stay due to healthcare-associated infections before and after implementation of a hospital-wide infection control program
Source: PLoS One. 2019 May 17;14(5):e0217159. doi: 10.1371/journal.pone.0217159 (PMC6524816; doi:10.1371/journal.pone.0217159)
Supplement: S1 Fig — (PDF) [file pone.0217159.s001.pdf]

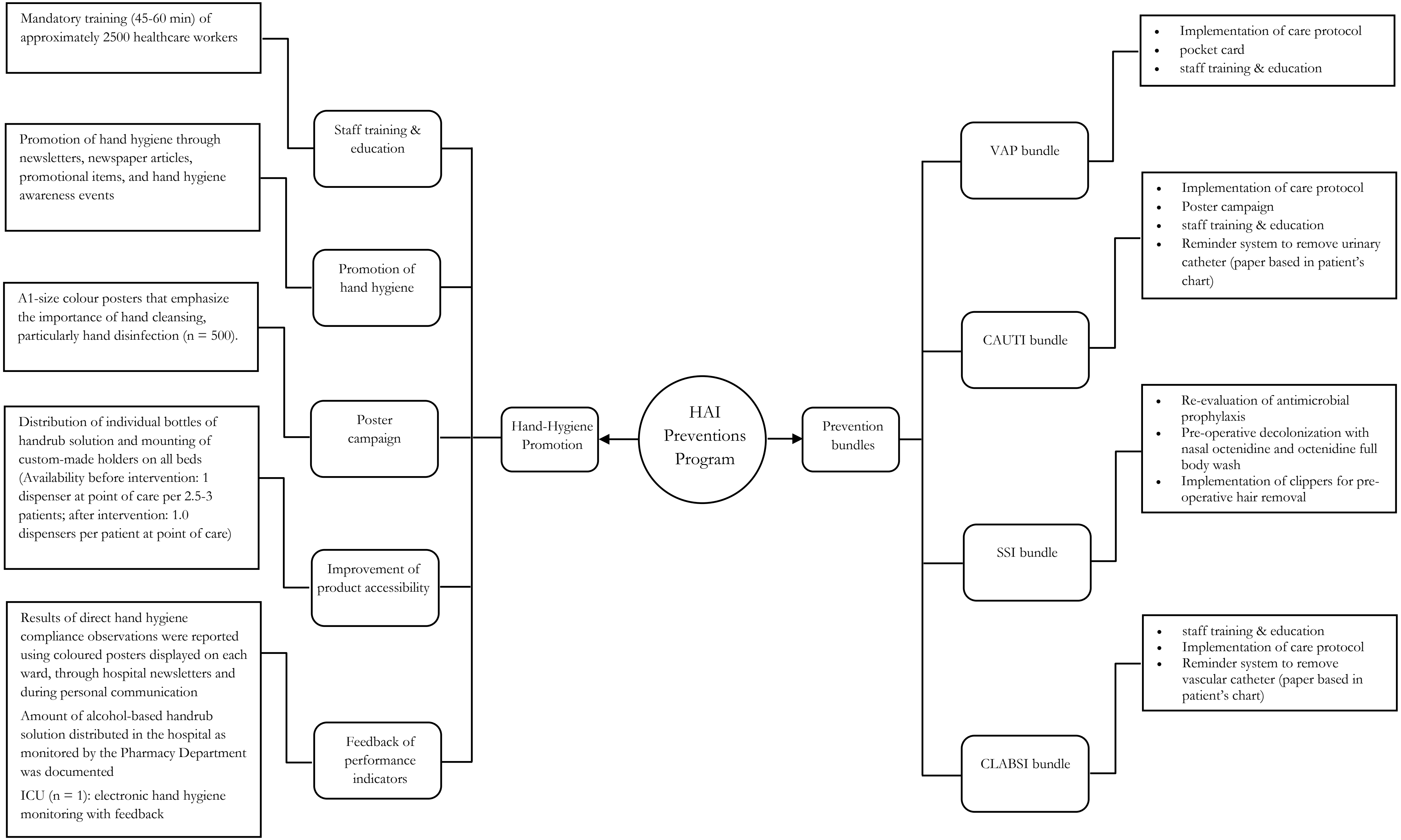

S1 Fig. Characterization of interventions for infection control in the ALERTS.

VAP, ventilator associated pneumonia; CAUTI, catheter-related urinary tract infection; SSI, surgical site infection; CLABSI, central line-associated bloodstream infections; ICU, intensive care unit.
