# Supplementary material for: Estimating extra length of stay due to healthcare-associated infections before and after implementation of a hospital-wide infection control program
Source: PLoS One. 2019 May 17;14(5):e0217159. doi: 10.1371/journal.pone.0217159 (PMC6524816; doi:10.1371/journal.pone.0217159)
Supplement: S2 Fig — (PDF) [file pone.0217159.s002.pdf]

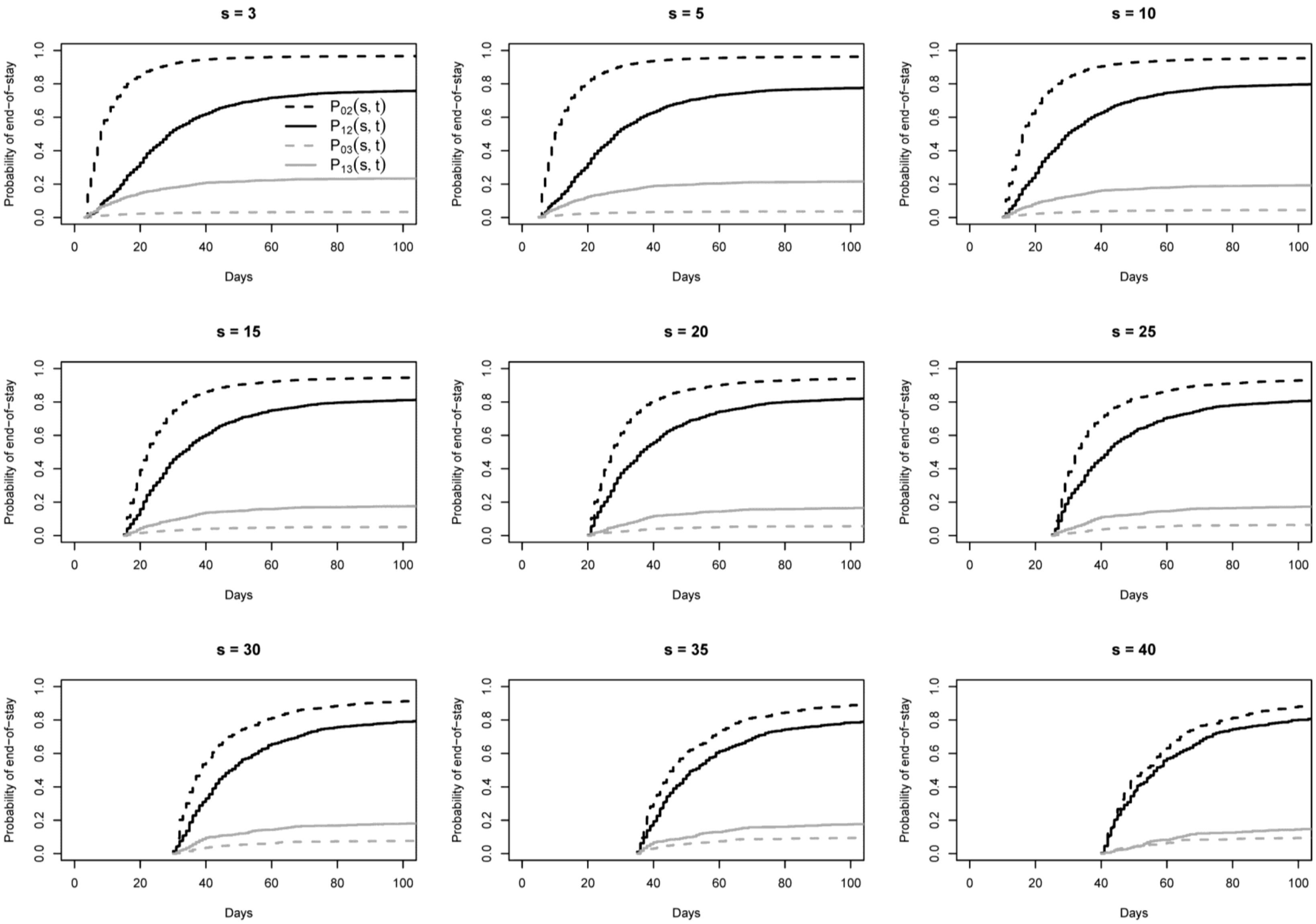

S2 Fig. Results of the Aalen-Johansen estimator for patients discharged alive [P02(s, t) (black dashed lines) and P12(s, t) (black solid lines)] and for death [P03(s, t) (dark grey dashed lines) and P13(s, t) (dark grey solid lines)] for different landmark times s in surveillance period 2.
